# Adulterations of Various Substances Used in Medicine and the Arts, with the Means of Detecting Them; Intended as a Manual for the Physician, the Apothcary, and the Artisan

**Published:** 1846-12

**Authors:** 


(For sale by Brautigam & Keen, Chicago.)

ARTICLE IX.

*Adulterations of various substances used in Medicine and the Arts, with the means of detecting them; intended as a manual for the Physician, the Apothcary, and the Artisan.* By LEWIS C. BECK, M. D., Professor of Chemistry in Rutger's College, New Jersey, and in the Albany Medical College, Honorary Member of the Medical Society of the State of New York, etc. New York: Samuel S. and William Wood.

1846. 12 mo. pp. 333. From the publishers. (For sale by Brautigam & Keen, Chicago.)

The subject upon which this treats is one of much interest to the apothecary and physician, and is generally too much neglected. The adulteration of medicines is carried on to such an extent, that all those who deal in them, should possess that knowledge necessary to distinguish between impure and genuine agents. This work is well calculated to impart such instruction; and the distinguished author, who is every way qualified to do justice to the subject, should be a sufficient recommendation. It should be in the library of every apothecary and physician. J. McL.

